# Array Comparative Genomic Hybridization (aCGH) Results among Patients Referred to Invasive Prenatal Testing after First-Trimester Screening: A Comprehensive Cohort Study

**DOI:** 10.3390/diagnostics14192186

**Published:** 2024-09-30

**Authors:** Anna Wójtowicz, Katarzyna Kowalczyk, Katarzyna Szewczyk, Anna Madetko-Talowska, Wojciech Wójtowicz, Hubert Huras, Mirosław Bik-Multanowski, Nowakowska Beata

**Affiliations:** 1 Department of Obstetrics & Perinatology, Jagiellonian University Medical College, 31-501 Kraków, Poland; hubert.huras@uj.edu.pl; 2 Department of Medical Genetics, Institute of Mother and Child, 30-663 Warsaw, Poland; katarzyna.kowalczyk@imid.med.pl (K.K.); beata.nowakowska@imid.med.pl (N.B.); 3 Department of Medical Genetics, Jagiellonian University Medical College, 30-551 Kraków, Poland; katarzyna.szewczyk@uj.edu.pl (K.S.); amadetko@usdk.pl (A.M.-T.); miroslaw.bik-multanowski@uj.edu.pl (M.B.-M.); 4 Information Technology Systems Department, Faculty of Management and Social Communication, Jagiellonian University, 30-348 Kraków, Poland; wojciech.wojtowicz@uj.edu.pl

**Keywords:** aCGH, array comparative genomic hybridization, chromosomal microarray, CMA, CNV, copy number variant, fetus, NT, nuchal translucency, prenatal diagnosis, ultrasound

## Abstract

**Introduction:** Invasive prenatal testing with chromosomal microarray analysis after first-trimester screening is a relevant option but there is still debate regarding the indications. Therefore, we evaluated the prevalence of numerical chromosomal aberrations detected by classic karyotype and clinically relevant copy number variants (CNVs) in prenatal samples using array comparative genomic hybridization (aCGH) stratified to NT thickness: <the 95th percentile, the 95th percentile–2.9 mm, 3.0–3.4 mm, 3.5–3.9 mm, 4.0–4.5 mm, and >4.5 mm, and by the presence/absence of associated structural anomalies detected by ultrasonography. **Materials and Methods:** Retrospective cohort study carried out at two tertiary Polish centers for prenatal diagnosis (national healthcare system) in central and south regions from January 2018 to December 2021. A total of 1746 prenatal samples were received. Indications for invasive prenatal testing included high risk of Down syndrome in the first-trimester combined test (n = 1484) and advanced maternal age (n = 69), and, in 193 cases, other reasons, such as parental request, family history of congenital defects, and genetic mutation carrier, were given. DNA was extracted directly from amniotic fluid (n = 1582) cells and chorionic villus samples (n = 164), and examined with classic karyotype and aCGH. **Results:** Of the entire cohort of 1746 fetuses, classical karyotype revealed numerical chromosomal aberrations in 334 fetuses (19.1%), and aCGH detected CNV in 5% (n = 87). The frequency of numerical chromosomal aberrations increased with NT thickness from 5.9% for fetuses with NT < p95th to 43.3% for those with NT > 4.5 mm. The highest rate of numerical aberrations was observed in fetuses with NT > 4.5 mm having at least one structural anomaly (50.2%). CNVs stratified by NT thickness were detected in 2.9%, 2.9%, 3.5%, 4.3%, 12.2%, and 9.0% of fetuses with NT < 95th percentile, 95th percentile–2.9 mm, 3.0–3.4 mm, 3.5–3.9 mm, 4.0–4.5 mm, and >4.5 mm, respectively. After exclusion of fetuses with structural anomalies and numerical aberrations, aCGH revealed CNVs in 2.0% of fetuses with NT < 95th percentile, 1.5% with NTp95–2.9 mm, 1.3% with NT 3.0–3.4 mm, 5.4% with NT 3.5–3.9 mm, 19.0% with NT 4.0–4.5 mm, and 14.8% with NT > 4.5 mm. **Conclusions:** In conclusion, our study indicates that performing aCGH in samples referred to invasive prenatal testing after first-trimester screening provides additional clinically valuable information over conventional karyotyping, even in cases with normal NT and anatomy.

## 1. Introduction

Screening for aneuploidy between 11 and 13^+6^ weeks of gestation has been a standard of prenatal care since the 1990s [1], and nuchal translucency (NT) is the single most effective ultrasound marker of chromosomal defects [2]. Valid values for NT depend on crown–rump length (CRL) and are those between the 5th and 95th percentiles [2]. The 95th percentile of NT increases linearly with CRL from 2.1 mm at a CRL of 45 mm to 2.7 mm for a CRL of 84 mm, whereas the 99th percentile does not change with CRL, and it is ~3.5 mm [2]. Studies have shown that increased NT alone or in combination with other ultrasound or serum biochemical markers is an effective tool for identifying trisomy 21. Moreover, NT may indicate a wide range of other genetic syndromes, major structural defects, risk of fetal death, miscarriage, and/or neurodevelopmental disorders [2,3,4,5]. Thus, there is consensus that NT ≥ the 99th percentile (≥3.5 mm) and/or the presence of additional structural defects are the indications for invasive prenatal testing [6,7,8,9,10]. However, there is still a debate regarding the management of NT values between the 95th and 99th percentile for gestational age and/or the so-called intermediate risk in the combined test. Currently, the prevailing opinion is that in the absence of a structural defect, it is an indication for cell-free DNA (cfDNA) analysis [9,11]. Undoubtedly, cfDNA is a highly effective test for trisomy 21 screening [12,13]; however, analysis of cfDNA does not show many other chromosomal aberrations of the fetus, such as microdeletions or microduplications, commonly referred to as copy number variants (CNVs) [14,15]. These submicroscopic aberrations are not detected in the classic karyotype but by chromosomal microarray analysis (CMA), which includes single nucleotide polymorphism (SNP) array and array comparative genomic hybridization (aCGH), the most commonly used chromosome microarray analysis. CMA identifies pathogenic chromosomal aberrations with more than 100 times greater resolution than classical karyotype testing [16]. Thus, currently, aCGH is recommended as a first-line genetic test for the prenatal evaluation of fetuses with structural anomalies observed by ultrasound and/or NT ≥ 3.5 mm [17,18]. CNVs can also have a major impact on the child’s neurological development; therefore, the prenatal assessment of the presence of pathogenic CNVs can have a significant impact on prognosis, the decision-making process of the parents, and health care planning. This information can also be helpful to parents in terms of enabling them to make an informed decision of whether to undergo invasive or noninvasive prenatal genetic testing.

Therefore, the aim of this study was to comprehensively evaluate the prevalence of copy number variants (CNVs) in prenatal samples using array comparative genomic hybridization (aCGH) stratified to NT thickness and presence/absence of structural anomalies.

## 2. Materials and Methods

This was a retrospective cohort study carried out at tertiary Polish centers for the prenatal diagnosis and management of fetal and neonatal pathology—the Department of Obstetrics and Perinatology in Cracow, the Department of Medical Genetics, Jagiellonian University Medical College in Cracow, and Department of Medical Genetics, Institute of Mother and Child in Warsaw. These centers have been conducting screening for aneuploidy for patients from central, southern, and eastern Poland since 2004, established by the Polish National Health Fund.

Databases were searched for cases undergoing invasive prenatal testing with known NT values after first-trimester screening between January 2018 and December 2021, where classical karyotype and aCGH were offered simultaneously. A total of 1746 samples meeting the inclusion criteria were recorded during the analyzed period.

First-trimester screening was carried out following Polish and international guidelines [19,20]. NT measurements were conducted according to the Fetal Medicine Foundation protocol [2] by physicians certified by the Fetal Medicine Foundation and Polish Society of Obstetrics and Gynecology.

The Polish protocol for first-trimester screening for aneuploidy is based on combined testing [7,10,19]. The individual risk for trisomy 21, 18, or 13 is based on maternal age, the fetal nuchal translucency (NT) value, and maternal serum markers, namely, free beta-hCG and PAPP-A. Other ultrasound parameters, such as the fetal nasal bone, tricuspid valve flow, blood flow in the ductus venosus, and fetal anatomy, were also assessed. NT ≥ 99th percentile (3.5 mm) and/or detection of structural anomalies are indications for invasive prenatal testing [10,19]. According to the Polish screening protocol [10], NT ≥ 3.5 mm is also an indication for referral to a tertiary-care center for further investigation in the form of additional ultrasound scans and echocardiography.

When NT thickness is above the 95th percentile but below 3.5 mm, the recommendation for invasive testing is based on risk calculated by the combined test (CT). The combined test high-risk cut-off used in Poland is >1:300, and in such cases, invasive testing is recommended. In cases with intermediate risk based on combined testing (1/300 to 1/1000) and without associated structural anomalies, the Polish guidelines recommended noninvasive prenatal testing with cfDNA tests, except in cases when the parents opt for prenatal invasive testing. In addition, in Poland, the provision of information about all diagnostic possibilities to patients is required, and therefore, each patient, regardless of the risk, is informed about the possibility of cfDNA as a screening method; however, these tests are not refunded by the Polish National Health Fund.

**Figure 1:**
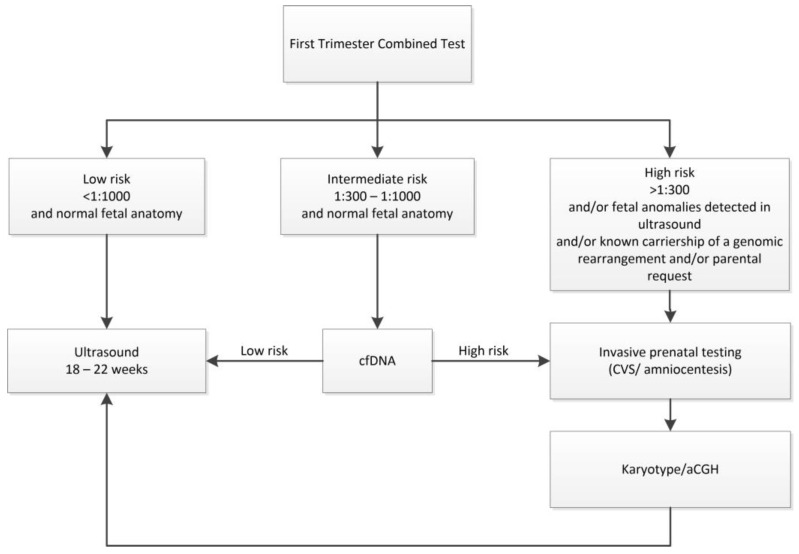
Prenatal diagnosis algorithm according to the Polish recommendations.

Fetuses with CT risk lower than 1/1000 are referred for second-trimester screening based on ultrasound [10]. Polish recommendations offer invasive testing not only for those patients with a positive screening result, but also in cases of a known carriership of a genomic rearrangement or an early onset genetic disease, pathogenic copy number variant (CNV) in previous pregnancy, and carriership of a pathogenic CNV or a balanced translocation in a parent, as well as in cases of parental anxiety (Figure 1).

Depending on the indication and gestational age, genetic testing was performed on chorionic villus samples (n = 164) or amniotic fluid (n = 1582).

The frequency of chromosomal aberrations was estimated for the following nuchal translucency categories: below the 95th percentile for CRL, between the 95th percentile for CRL and 2.9 mm, 3.0–3.4 mm, 3.5–3.9 mm, 4.0–4.5 mm, and >4.5 mm. The analyses were also conducted in each category in relation to the fetuses, where the anatomy assessed by ultrasound in the first and early second trimesters was normal or not.

Informed consent for genetic studies was obtained from all pregnant women, and pre-test and post-test counseling, including provision of information about the risks associated with the procedure and the diagnostic techniques involved, was performed by trained clinical geneticists. Those who chose to participate provided written informed consent after the discussion of the potential advantages and risks of chromosomal microarray testing, including the possibility of identifying variants of uncertain clinical significance (VOUSs) and genetic variants in the fetus that cause adult-onset disorders.

### 2.1. Genetic Testing

#### Array Comparative Genomic Hybridization Analysis and Interpretation

Genetic testing with the aCGH procedure was performed at the Department of Medical Genetics, Jagiellonian University Medical College, and the Department of Medical Genetics, Institute of Mother and Child, Warsaw, as previously described in detail [21,22] following amniocentesis (n = 1582) or chorionic villus sampling (n = 164).

Unprocessed amniotic fluid (~10 mL) or chorionic villi were used for genomic DNA extraction using the Genomic Mini AX Body Fluids kit (A&A Biotechnology, GdańskPoland) or QuickGene DNA tissue kit S (Kurabo Industries, Osaka, Japan). DNA concentration and purity were determined using a NanoDrop 1000 spectrophotometer (Thermo Scientific, Waltham, MA, USA). aCGH analysis was performed with 8 × 60 K oligonucleotide microarrays (Agilent, Santa Clara US or Oxford Gene Technology, Cytosure, Oxfordshire, UK) following the manufacturer’s protocol.

The results were reported in a specific consultation for post-test genetic counseling, where the results and implications crucial to support informed decision-making were explained to the women.

Results of a pathogenic or potentially pathogenic nature were revealed to patients, with subsequent comprehensive genetic counseling given. Additionally, in cases with fetal pathogenic CNVs, parents were encouraged to undergo aCGH to verify if the fetal CNVs were inherited or appeared de novo.

The aCGH procedure and interpretation were conducted as described previously [21,22]. The reported CNVs were classified according to European standards [21]. A newly identified CNV was considered a relevant chromosomal aberration if it was detected in a minimum of 3 consecutive oligonucleotide microarray probes. Aberrations were filtered up to a minimal size of 1 Mb for deletions and duplications. However, CNVs smaller than 1 Mb were included for further analysis if they were clearly pathogenic or potentially pathogenic based on data from the following databases: ClinVar (Clinically Relevant Variation Database; https://www.ncbi.nlm.nih.gov/clinvar/ (accessed on 5 May 2024)); DECIPHER (Database of Chromosomal Imbalance and Phenotype in Humans Using Ensembl Resource, http://decipher.sanger.ac.uk/ (accessed on 28 April 2024)); databases supported by data from OMIM (Online Mendelian Inheritance in Man: http://www.ncbi.nlm.nih.gov/omim (accessed on 28 April 2024)); the UniQue databases; the Database of Genomic Variants (DGV, http://dgv.tcag.ca/dgv/ (accessed on 29 April 2024)); Clinical Genome Resource (ClinGen, https://www.clinicalgenome.org/ (accessed on 5 May 2024)); and PubMed (https://www.ncbi.nlm.nih.gov/pubmed/ (accessed on 5 May 2024)). The CNVs were assigned the following interpretations according to international guidelines [18,23,24,25]: (1) pathogenic or likely pathogenic, (2) variants of uncertain significance (VUSs), and (3) benign and likely benign. In our study, we focused on pathogenic/likely pathogenic CNVs.

This study was approved by the Medical Ethical Committee of the Medical College Jagiellonian University of Kraków (KBE1072.6120.248.2021).

### 2.2. Statistical Analysis

All data were recorded in an electronic database. Any direct identifiers were removed and replaced by a code. Patient characteristics are described as means with standard deviation for normally distributed numerical data and as percentages for categorical variables. Difference in maternal age was analyzed by the Student *t*-test. Chi-square and Fisher’s exact tests were used for comparisons of categorical variables. In all analyses, *p* values < 0.05 were considered statistically significant. SPSS 19.0 software was used for statistical analysis.

## 3. Results

In 1484 (84.9%) cases, invasive prenatal diagnosis was indicated because of abnormal results from combined first-trimester screening. In 69 (3.9%) cases, invasive tests were indicated because of advanced maternal age, and in 193 (11.2%) cases, other reasons, such as parental request, family history of congenital defects, and genetic mutation carrier, were given.

In the entire cohort of 1746 fetuses who underwent invasive prenatal testing, classical karyotype testing revealed numerical chromosomal aberrations in 334 fetuses (19.1%), and aCGH detected clinically significant CNVs in 87 (5%) additional cases (Table 1). The most common numerical aberration was trisomy 21 (n = 174), and 22q11.2 microdeletion was the most common CNV, occurring in 21 cases. The sizes of CNVs ranged from 0.01 Mb to 93.4 Mb (Table 2). In 608 fetuses, at least one structural anomaly was detected by ultrasound. The most common anomalies were cardiovascular defects, which occurred in 210 (12.0%) cases. Among the ultrasound markers assessed in the first trimester, the most common finding was nasal bone hypoplasia, which was detected in 10.1% (177/1746) of fetuses (Table 1).

In 754 (43.2%) cases, NT was below the 95th percentile, and in 992 (56.8%) cases, NT was ≥95th percentile (Table 1). In the subgroup of fetuses with NT < p95, numerical chromosomal aberrations were detected in 45 cases (5.9%) (Table 1) and CNVs in 22 (2.9%) cases (Table 1). In 39.8% of cases, there was one or more additional structural anomalies, and the most common ultrasound findings were cardiovascular anomalies (13.5%), followed by nasal bone hypoplasia (11.7%) and skeletal anomalies (8.2%) (Table 1). In 454 (60.2%) fetuses with NT < the 95th percentile and without associated structural anomalies detectable during first- or early second-trimester ultrasound screening, chromosomal aberrations were revealed in 5.3% (Table 1), with trisomy 21 and structural CNVs occurring with the same frequency of 1.9%.

In 992 (56.8%) patients, NT was ≥p95 (Table 1), with 621 (35.5%) fetuses having NT ≥ 3.5 mm. Numerical chromosomal aberrations were detected in 29.1% of fetuses with NT ≥ p95, and CNV in 6.5% of cases (Table 1). The most common numerical aberration was trisomy 21 (15.8%), followed by Turner syndrome (5.7%) and trisomy 18 (4.8%) (Table 1). The three most common trisomies accounted for 63.3% of all pathogenic chromosomal aberrations. In the cohort of fetuses with NT ≥ p95 and after excluding numerical aberrations, CNVs were present in 9.2% of fetuses (65/703). 22q21 microdeletions/microduplications were the most common among CNVs (21.5%; 14/65) (Table 2). In 684 cases (68.9%) of fetuses with NT ≥ p95th, no associated structural anomalies were noted, and 31.1% had at least one associated structural defect. The most common were cardiovascular anomalies (10.8%), followed by hydrops (8.1%). Similar to the group of fetuses with NT < p95th, the most common first-trimester ultrasound marker was NB hypoplasia (8.9%) (Table 1).

In the subgroup of fetuses with NT ≥ 3.5 mm, pathogenic chromosomal aberrations were present in 44.7% (278/621). In 36.2% (225/621) of cases, karyotype revealed numerical chromosomal aberrations and aCGH detected CNVs in 8.5% (53/621) of cases.

Considering the ranges of NT values, the frequency of numerical chromosomal aberrations increased with NT thickness from 5.9% for fetuses with NT < p95th to 43.3% for those with NT > 4.5mm (Table 2). Regarding CNV incidence, no significant differences were observed between fetuses with NT < p95 and those with NT values up to 3.5mm (Table 2). The highest percentage of CNVs was observed in the group of fetuses with NT 4.0–4.5mm (12.2%) (Table 3).

**Figure 2:**
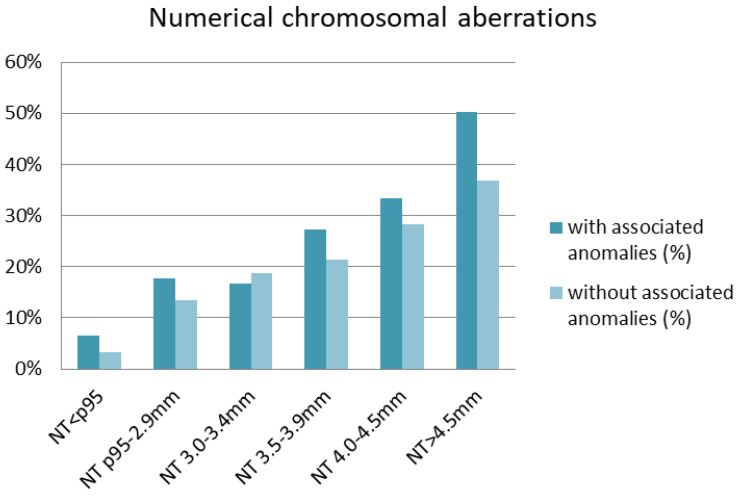
Distribution of numerical chromosomal aberrations depending on nuchal translucency (NT) thickness.

**Figure 3:**
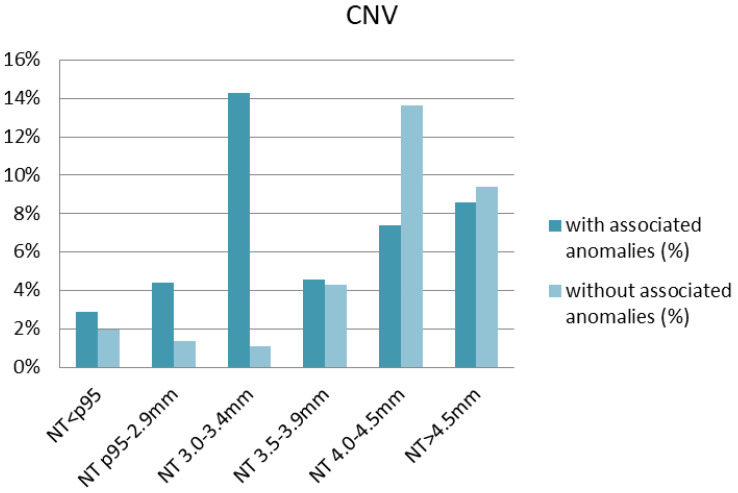
Distribution of copy number variants (CNVs) depending on the nuchal translucency (NT) thickness.

The studied cohort was also stratified according to NT thickness and the presence or absence of accompanying anomalies (Table 4). Both in the absence and presence of accompanying anomalies, the frequency of detected numerical aberrations increased with NT thickness (Figure 2). The highest rate of numerical aberrations was observed in fetuses with NT > 4.5 mm having at least one structural anomaly (50.2%). With regard to CNVs, no significant differences in the frequency were observed between fetuses with NT < p95 and those with NT p95-2.9 mm (Table 4). CNVs occurred most frequently in fetuses with NT 3.0–3.4 mm (14.3%) in which additional structural anomalies were diagnosed, followed by fetuses with NT 4.0–4.5 mm and without accompanying anomalies (13.6%) (Figure 3). Interestingly, in fetuses with a normal karyotype and NT < p95th, microarray analysis revealed clinically relevant deletions or duplications in 4.8% with a structural anomaly and in 2.0% of those without structural defects (Table 4). After excluding cases with structural anomalies and numerical aberrations, CNVs occurred in 1.3% of fetuses with NT p95th-3.4 mm and in 13.0% with NT ≥ 3.5 mm.

## 4. Discussion

This was a retrospective study analyzing the results of classical karyotype and aCGH depending on NT thickness and the occurrence/absence of associated defects.

In the entire cohort of 1746 fetuses studied, 19.1% of numerical aberrations were detected and aCGH additionally revealed CNVs in 5% of fetuses.

The frequency of numerical aberrations increased with NT thickness and in the case of additional defects. In the subgroup of fetuses with NT > 4.5 mm and at least one anomaly, numerical chromosomal aberration was detected in 50.2% cases. Clinically significant microdeletions/microduplications were present in each analyzed subgroup, with the highest percentage in the group of fetuses with NT 3.0–3.4 mm having at least one structural anomaly.

In recent years, the association between increased NT and pathogenic CNVs has been extensively explored in the literature [9]. However, there is still a debate about the NT value at which we should refer patients for invasive diagnostics or recommend cfDNA testing, especially when increased NT is not accompanied by structural abnormalities or there is increased risk of aneuploidy after the first-trimester combined test [12,26]. It is common for physicians conducting first-trimester screening for aneuploidy to have doubts about the choice regarding next steps in the diagnostic path for patient with a NT between the 95th percentile and 3.5 mm, and those with NT within the normal range but the combined test indicates a high risk of aneuploidy. We believe that the results of our research will help to make decisions about further diagnostic steps.

Currently, NT ≥ 3.5 mm is an undisputed indication for invasive prenatal testing. However, the percentage of detected chromosomal aberrations varies between studied cohorts. The rate of chromosomal abnormalities in fetuses with NT ≥ 3.5 mm (44.7%) was higher in our study than that reported by Srebniak et al. (38%) [14] but similar to that reported by Bardi et al. (43%) [27]. It was reported in the literature that pathogenic CNVs can be detected by CMA in 0–15% of fetuses with NT ≥ 3.5 mm depending on the selected or unselected nature of the studied cohorts [9,15,28,29,30]. In our study, pathogenic CNVs were detected in 8.5% of cases with NT ≥ 3.5 mm in the unselected population and in 13.0% of cases with isolated NT ≥ 3.5 mm where karyotype and anatomy were assessed as normal. These results are similar to those reported by Tanner et al. (13.7%) [31] and by Coello-Cahuao et al. (12%) [32]. However, in other studies [9,27], the frequency of pathogenic CNVs among fetuses with normal karyotype and isolated increased NT ≥ 3.5 mm was reported to be between 3% and 9%. In our study, the frequency was also higher than that in another French multicenter project [33], where it was 2.7%, but in the French study, fetuses between 11 and 28 weeks of gestation were included. It could be supposed that in our study, we did not observe all structural anomalies, as the study was conducted during the first- and early second-trimester. Therefore, when giving a consultation, the duration of pregnancy should be considered. It can be assumed that some structural abnormalities may appear in later stages of pregnancy; on the other hand, in some cases of fetal structural and/or genetic defects, stillbirth may occur in the following weeks.

Recently, a cut-off of 3.0 mm was proposed as an indication for CMA [11,34]. In previous studies where fetuses with NT between 3.0 and 3.4 mm were included, the prevalence of CNVs varied between 0.4 and 2.1% [34,35,36]. In our cohort, it was 4.3% in an unselected cohort with a normal karyotype and in 1.3% of fetuses with a normal karyotype and normal anatomy.

Moreover, in our study, 21.5% of chromosomal aberrations were found in pregnancies with NT values between the 95th and 99th percentiles, which is similar to the observation by Bardi et al. [27]. On the other hand, in our study, CNVs were present in 1.4% of fetuses with NT p95th–99th percentile with normal karyotypes, and in previous studies, this prevalence was estimated to be between 1.7% and 2.6% [37,38].

In our study, chromosomal aberrations were diagnosed in 35.7% of the fetuses with NT ≥ 95th percentile, which is higher than previously reported [27]. A total of 22.5% of fetuses with NT above the 95th percentile had trisomy 21, 13, or 18, which is similar to the finding by Bardi et al. [27], where these three common trisomies were reported in 23.9% of fetuses.

It was shown that in the absence of aneuploidy, fetuses with increased NT have an increased risk of other fetal defects, particularly cardiac and skeletal abnormalities [5,22].

In our study, 29.6% of fetuses with NT p95th–99th percentiles and 31.9% of fetuses with NT above the 99th percentile were found to have at least one structural anomaly, and in the study by Bardi et al. [27], the percentages were 21.3% and 62%, respectively.

In their prospective cohort study, Wapner et al. [16] reported that in pregnancies with fetal structural anomalies and a normal karyotype, the incremental diagnostic yield with CMA was approximately 6% above what a karyotype could detect. These authors [16] also reported a 1.7% diagnostic yield of CMA in samples with normal conventional karyotype when the indication was advanced maternal age or positive screening results. In our observation, CNVs were detected in 2.0% of fetuses with NT < the 95th percentile where karyotype and anatomy were normal. However, a recent meta-analysis [39] indicated that 0.84% of fetuses that had invasive testing because of advanced maternal age or anxiety carried a pathogenic clinically significant submicroscopic aberration, and that pregnant women under 36 years of age have a higher risk for submicroscopic pathogenic aberrations than for trisomy 21.

Moreover, it should be noted that, according to the last study [40], even when the karyotype and microarray are normal, there is an increased residual risk of morbidity-related outcomes compared with the general population, particularly if NT is greater than 6 mm.

### Strengths and Limitations

The strength of this study is that it was a two-center cohort study that included a representative study population due to publicly funded prenatal care. This allowed us to evaluate the frequency of chromosomal aberrations and CNVs in subgroups with different NT thickness, which may be helpful for clinicians to choose the most appropriate test. The limitation is that it was a retrospective study, and we did not have a long follow-up, but only the data from the first- and early second-trimester scans. Therefore, we did not analyze all pregnancy outcomes, and we therefore cannot estimate the actual impact of the results on the outcome. Second, in some cases, it is possible that some genetic disorders might have been detected if other genetic methods had been used.

## 5. Conclusions

In fetuses undergoing invasive prenatal testing because of increased NT and/or increased risk after first-trimester screening, microarray analysis should be considered as the method of choice, as it allows the detection of submicroscopic anomalies that are not detected by karyotyping alone, even in the absence of structural abnormalities. Increased NT ≥ 3.5 mm is a strong indication for genetic testing with the aCGH technique. However, according to our results, in cases with NT <the 99th percentile and normal anatomy during the first trimester, aCGH can detect pathogenic CNVs in a significant percentage of cases.

Therefore, invasive testing with aCGH should be discussed with parents choosing invasive procedures.

Key Message: Performing aCGH in fetuses referred for invasive prenatal testing after first-trimester screening provides additional, clinically valuable information compared to conventional karyotyping, even in the presence of normal anatomy. This information can be helpful to parents, enabling them to make an informed decision about which diagnostic path to take.

## Figures and Tables

**Table 1:** Nuchal translucency (NT) and associated ultrasound and genetic findings among 1746 fetuses undergoing invasive prenatal testing.

	NT < p95 n = 754	NT ≥ p95 n = 992	OR (95% CI)	*p*
Maternal age, years				
mean ± SD	34.98 ± 5.1614	35.5 ± 5.5	-	0.326
Min–max	17–46	18–47		
median	35	36		
All pathogenic chromosomal aberrations, n (%)	67 (8.8)	354 (35.7)	5.7 (7.54–4.28)	0.0000
Numerical chromosomal aberrations (total), n (%)	N = 45 (5.9%)	N = 289 (29.1%)	6.48 (4.66–9.02)	0.0000
T21, n (%)	17 (2.3)	157 (15.8)	8.15 (4.89–13.57)	0.0000
T18, n (%)	7 (0.9)	48 (4.8)	5.4 (2.44–12.06)	0.0000
T13, n (%)	5 (0.6)	19 (1.9)	2.925 (1.08–7.87)	0.026
45, X0, n (%)	3 (0.4)	57 (5.7)	15.26 (4.76–48.92)	0.0000
47, XXX; 47, XXY, n (%)	5 (0.6)	5 (0.5)	0.75 (0.21–2.63)	0.66
Triploidy, n (%)	8 (1.0)	3 (0.3)	0.34 (0.11–1.11)	0.06
CNV, n (%)	22 (2.9)	65 (6.5)	2.33 (1.42–3.81)	0.0005
Associated structural anomalies				
-none, n (%)	454 (60.2)	684 (68.9)	1.46 (1.20–1.78)	0.0001
-one or more anomalies, n (%)	300 (39.8)	308 (31.1)	0.68 (0.83–0.56)	0.0001
Structural anomalies				
-CNS, n (%)	55 (7.3)	14 (1.4)	0.18 (0.1–0.32)	0.0000
-cardiovascular anomalies, n (%)	102 (13.5)	108 (10.8)	0.78 (0.58–1.04)	0.092
-genitourinary tract, n (%)	20 (2.6)	16 (1.6)	0.60 (0.30–1,16)	0.1300
-gastro-abdominal, n (%)	40 (5.2)	13 (1.3)	0,23 (0.12–0.44)	0,0000
-skeletal, n (%)	62 (8.2)	33 (3.3)	0.38 (0.24–0.59)	0,0000
-face/neck, n (%)	35 (4.6)	36 (3.6)	0.77 (0.48–1.24)	0.2885
-pulmonary/thoracic, n (%)	11 (1.4)	6 (0.6)	0.41 (0.15–1.11)	0.07
-oedema, n (%)	4 (0.5)	81 (8.1)	16.6 (6.08–45.70)	0.0000
-FGR, n (%)	39 (5.1)	6 (0.6)	0.11 (0.04–0.26)	0.0000
-amniotic fluid anomaly, n (%)	17 (2.2)	3 (0.3)	0.13 (0.03–0.45)	0.0001
First-trimester markers:				
-TR, n (%)	39 (5.1)	52 (5.2)	1.01 (0.66–1.55)	0.94
-DV abnormal, n (%)	33 (4.4)	34 (3.4)	0.77 (0.47–1.26)	0.30
-NB hypoplasia, n (%)	88 (11.7)	89 (8.9)	0.74 (0.54–1.01)	0.064
Fetuses without associated anomalies, n (%)	N = 454 (60.2)	N = 684 (68.9)	1.46 (1.20–1.78)	0.0001
All pathogenic chromosomal aberrations, n (%)	24 (5.3)	224 (32.7)	8.72 (5.61–13.56)	0.0000
Numerical chromosomal aberrations (total), n (%)	15 (3.3)	184 (26.9)	10.77 (6.26–18.51)	0.0000
T21, n (%)	9 (1.9)	123 (18.0)	10.84 (5.44–21.57)	0.0000
T18, n (%)	0 (0.0)	20 (2.9)	-	0.0002
T13, n (%)	0 (0.0)	5 (0.7)	-	0.0679
45, X0 n (%)	2 (0.4)	32 (4.7)	0.03 (0.01–0.16)	0.0000
47,XXX; 47,XXY, n (%)	4 (0.8)	4 (0.6)	0.66 (0.16–2.65)	0.5580
Triploidy, n (%)	0 (0.0)	0 (0.0)	-	-
CNV, n (%)	9 (1.9)	40 (5.8)	3.07 (1.47–6.39)	0.0017

aCGH, array comparative genomic hybridization; bCNV, benign copy number variants; CNS, cerebral nervous system; CNV, copy number variants; DV, ductus venosus; FGR, fetal growth restriction; NB, nasal bone; NT, nuchal translucency; pCNV, pathogenic/likely pathogenic copy number variants; SD, standard deviation; TR, tricuspid valve regurgitation; T13, trisomy 13; T18, trisomy 18; T21, trisomy 21; VOUS, variants of unknown significance.

**Table 2:** Pathogenic or likely pathogenic copy number variants identified by array comparative genomic hybridization (aCGH).

No	ISCN 2020 arr[GRCh37]	Size and Type	CNV Interpretation	Detailed Clinical Indications
1.	6q27(168157088_170041859)x1	1.88 Mbp deletion	Female karyotype, 6q microdeletion syndrome reported in UNIQUE database: https://www.rarechromo.org/media/information/Chromosome%20%206/6q%20deletions%20from%206q26%20and%206q27%20FTNP.pdf (accessed on 29 April 2024)	NT = 2 mm First-trimester abnormal biochemistry, high risk of Down syndrome
2.	10q22.3q23.2(81641918_88847443)x1	7.2 Mbp deletion	Male karyotype, genome imbalance was found in the form of a deletion of the long arm of chromosome 10 in the 10q22.3q23.2 region	NT = 3 mm High risk of Down syndrome Agenesis of one kidney
3.	22q11.21(18894820_21457610)x1	2.56 Mbp deletion	Female karyotype, genome imbalance was found in the form of an interstitial deletion of the long arm of chromosome 22 in the 22q11.21 region. The deletion covers the 22q11.2 deletion syndrome region (OMIM 188400)1 (ORPHA:567)2, including 52 protein-coding genes. Deletions of this region have been described in patients, among others, with heart defects, anatomical abnormalities of the palate, facial dysmorphic features, developmental delay, thymic aplasia/hypoplasia and immunodeficiency	NT = 3 mm High risk of Down syndrome, RAA
4.	Xq22.3(105159857_105621192)x2	460 kb duplication	Male karyotype, genome imbalance was found in the form of a duplication of the long arm of the X chromosome in the Xq22.3 band with a size of ~460 kb. The duplication includes the *SERPINA7* gene (OMIM 314200) and exons 16–29 of the *NRK* gene (OMIM 300791). Amplification of the *SERPINA7* gene has been described in patients with excess thyroxine-binding globulin (TBG).	NT = 4 mm High risk of Down syndrome
5.	5p15.33p11(22149_46115173)x3 dn	46.09 Mbp duplication	Female karyotype, in which genome imbalance was found in the form of the short arm of chromosome 5 duplication in the 5p15.33p11 region with a size of ~46.09 Mbp, which corresponds to trisomy of the short arm of chromosome 5. The aberration affects many genes and is a pathogenic change. Chromosome analysis after cell culture (DM700/19) showed the presence of two cell lines: mos46,XX[11]/47,XX,del(5)(p11),+i(5)(p11)[3]. In 3 out of 14 cells analyzed, a deletion of the short arm of chromosome 5 and the presence of an additional isochromosome 5p were found, and in the remaining 11 cells analyzed, a normal female karyotype was found. The presence of a normal female cell line may indicate the growth of cells of maternal origin in the resulting culture.	NT = 4 mm High risk of Down syndrome
6.	3q24q29(147103808_197837069)x2~3, 4q34.1q35.2(174521042_190791091)x1~2	50.73 Mbp duplication 16.27 Mbp deletion	Male karyotype, genome imbalance was found in the form of a mosaic duplication of the long arm of chromosome 3 in the 3q24q29 region of ~50.73 Mbp and a mosaic deletion of the long arm of chromosome 4 in the 4q34.1q35.2 region of ~16.27 Mbp. Aberrations involve many genes and may be responsible for abnormalities detected in the fetus during ultrasound examination.	NT = 4 mm High risk of Down syndrome
7.	6p21.33(31946615_32009011)x1	60 kb deletion	Male karyotype, genome imbalance was found in the form of a deletion of the short arm of chromosome 6 in the 6p21.33 region of ~60 kb. The deletion includes the following genes: C4A (OMIM 120810)1, C4B (OMIM 120820)1, CYP21A2 (OMIM 613815)1 and exons 4–8 of the *STK19* gene (OMIM 604977)1. Due to the family history of congenital adrenal hyperplasia, it is advisable to exclude the presence of mutations in the second allele of the *CYP21A2* gene.	NT = 4 mm High risk of Down syndrome, positive family history of congenital adrenal hyperplasia in daughter from a previous pregnancy.
8.	22q11.21(18894820_21457610)x1	2.56 Mb deletion	Male karyotype, genome imbalance was found in the form of an interstitial deletion of the long arm of chromosome 22 in the 22q11.21 region with a size of ~2.56 Mbp. The deletion covers the 22q11.2 deletion syndrome region (OMIM 188400)1 (ORPHA:567)2, including 52 protein-coding genes. Deletions of this region have been described in patients, among others, with heart defects, anatomical abnormalities of the palate, facial dysmorphic features, developmental delay, aplasia/hypoplasia of the thymus and immunodeficiency.	NT = 4 mm High risk of Down syndrome, CHD (TOF)
9.	22q11.21(18894820_21457610)x1	2.56 Mb deletion	Male karyotype, genome imbalance was found in the form of deletion of the long arm of chromosome 22 in the 22q11.21 region with a size of ~2.56 Mbp. The deletion is located in the region of the known 22q11 microdeletion syndrome (OMIM: 188400).	NT = 5 mm omphalocele,HLHS, NB hypoplasia High risk of Down syndrome
10.	22q11.21(18894820_21457610)x1	2.56 Mb deletion	Female karyotype, genome imbalance was found in the form of an interstitial deletion of the long arm of chromosome 22 in the 22q11.21 region with a size of ~2.56 Mbp. The deletion covers the 22q11.2 deletion syndrome region (OMIM 188400)1 (ORPHA:567)2, including 52 protein-coding genes. Deletions of this region have been described in patients, among others, with heart defects, anatomical abnormalities of the palate, facial dysmorphic features, developmental delay, aplasia/hypoplasia of the thymus and immunodeficiency.	NT = 5 mm High risk of Down syndrome, Abnormal profile
11.	22q11.21(18894820_21457610)x1	2.56 Mb deletion	Male karyotype, genome imbalance was found in the form of an interstitial deletion of the long arm of chromosome 22 in the 22q11.21 region with a size of ~2.56 Mbp. The deletion covers the 22q11.2 deletion syndrome region (OMIM 188400)1 (ORPHA:567)2, including 52 protein-coding genes. Deletions of this region have been described in patients, among others, with heart defects, anatomical abnormalities of the palate, facial dysmorphic features, developmental delay, aplasia/hypoplasia of the thymus and immunodeficiency.	NT = 5 mm High risk of Down syndrome HLHS
12.	13q14.2q34(49772419_115093115)x3	65.3 Mbp duplication	Male karyotype, genome imbalance was found in the form of a duplication of the long arm of chromosome 13 in the 13q14.2q34 region with a size of ~65.3 Mbp.	NT = 6 mm High risk of Down syndrome micrognatia, polidactyly
13.	14q11.2(21859914_21913304)x1	53 kb deletion	Male karyotype, genome imbalance was found in the form of an interstitial deletion of the long arm of chromosome 14 in the 14q11.2 region with a size of ~53 kb. The deletion covers exons 1–35 of the dose-sensitive *CHD8* gene (OMIM 610528)1 and is located in the region of the 14q11-q22 deletion syndrome (OMIM 613457)1 (ORPHA:261120)3, described in patients among others, with, delayed psychomotor development, hypotonia, facial dysmorphic features. Additionally, mutations and deletions of the *CHD8* gene have been described in patients, among others, with autism.	NT = 7 mm High risk of Down syndrome
14.	1p21.3p12(95352587_118623859)x1	23.3 Mbp deletion	Male karyotype, genome imbalance was found in the form of an interstitial deletion of the short arm of chromosome 1 in the 1p21.3p12 region with a size of ~23.3 Mbp. The deletion in the 1p21.3p12 region covers 157 protein-coding genes, including the following genes: DPYD (OMIM 612779)1, DBT (OMIM 248610)1, dose-sensitive COL11A1 (OMIM 120280)1, GPSM2 (OMIM 609245)1, dose-sensitive TAF13 (OMIM 600774)1, AP4B1 (OMIM 607245)1, NRAS (OMIM 164790)1 and CASQ2 (OMIM 114251)1. The aberration covers the region of the 1p21.3 microdeletion syndrome (ORPHA:293948)2 described in patients including those with speech development delay, intellectual disability, autism, facial dysmorphic features and obesity. Deletions in the 1p13.1p21.1 region have been described in patients, among others, with intellectual disability, short stature, dysmorphic features, and eye defects.	NT = 7 mm High risk of Down syndrome
15.	5q35.2q35.3(175116131_180696832)x1	5.58 Mbp deletion	Female karyotype, genome imbalance was found in the form of a terminal deletion of the long arm of chromosome 5 in the 5q35.2q35.3 region with a size of ~5.58 Mbp. The deletion covers 87 protein-coding genes, including the NSD1 gene (OMIM 606681) and is located in the region of 5q35 deletion syndrome (ORPHA:1627)2, characterized by: lymphedema with widening of the nuchal translucency In the prenatal period, as well as early infantile hypotonia, short stature, facial dysmorphic features and heart defects. The 5q35 deletion regionIdes the region of Sotos syndrome 1 (SOTOS1, OMIM 117550), described in patients with facial dysmorphic features, high birth weight and excessive growth in early life, macrocephaly, intellectual disability, as well as heart defects such as atrial septal defect (ASD), ventricular septal defect (VSD).	NT = 7 mm High risk of Down syndrome CHD (ASD)
16.	22q11.21(18894820_21457610)x1	2.56 Mbp deletion	Male karyotype, genome imbalance was found in the form of an interstitial deletion of the long arm of chromosome 22 in the 22q11.21 region with a size of ~2.56 Mbp. The deletion covers the 22q11.2 deletion syndrome region (OMIM 188400) (ORPHA:567), including 52 protein-coding genes. Deletions of this region have been described in patients, among others, with heart defects, anatomical abnormalities of the palate, facial dysmorphic features, developmental delay, aplasia/hypoplasia of the thymus, and immunodeficiency.	NT = 8 mm High risk of Down syndrome
17.	Xp21.1(31837550_32165393)x1,(21)x3	328 kb deletion	Female karyotype, genome imbalance was found in the form of trisomy of chromosome 21 and deletion of the short arm of the X chromosome in the Xp21.1 region of ~328 kb. Trisomy of chromosome 21 corresponds to the diagnosis of Down syndrome. The deletion in the Xp21.1 region covers exons 45–78 of the dose-sensitive DMD gene (OMIM 300377). Mutations and deletions of this gene have been described in patients with Duchenne muscular dystrophy (OMIM 310200), Becker muscular dystrophy (OMIM 300376) and dilated cardiomyopathy (OMIM 302045). The aberration is a pathogenic change and may be inherited from carrier mothers who do not show clinical symptoms.	NT = 9 mm High risk of Down syndrome
18.	3q26.32q26.33(177804563_180939724)x3	3.1 Mbp duplication	Male karyotype, 3q duplication syndrome reported in the UNIQUE database, phenotype depending on the size and location of the aberration https://www.rarechromo.org/media/information/Chromosome%20%203/3q%20duplications%20FTNP.pdf (accessed on 29 April 2024)	NT = 10 mm High risk of Down syndrome NB hypoplasia, flat profile, club foot
19.	18p11.32p11.21(149089_11050847)x1, 18p11.21q23(11162185_77794890)x3	11 Mbp deletion 66.7 Mbp duplication	Female karyotype, genome imbalance was found in the form of a deletion of the short arm of chromosome 18 in the 18p11.31p11.21 region of ~11 Mbp and a duplication in the 18p11.21q23 region of ~66.7 Mbp	NT= 10 mm, Multiple structural anomalies High risk of Down syndrome
20.	13q33.1q34(103658425_115092648)x1, 21q22.2q22.3(39764621_48090317)x3	11.4 Mbp deletion	Male karyotype, common phenotype after both changes: del13 CHROMOSOME 13q33-q34 DELETION SYNDROME (OMIM#619148) and dup21 genes which are essential in producing the main Down syndrome hen CP, Chen CY, Chern SR, et al. Detection of a familial 21q22.3 microduplication in a fetus associated with congenital heart defects. *Taiwan J. Obstet. Gynecol.* **2019**, *58*, 869–871 features cluster in chromosomal bands 21q22.2q22.3 Schnabel F, Smogavec M, Funke R, Pauli S, Burfeind P, Bartels I. Down syndrome phenotype in a boy with a mosaic microduplication of chromosome 21q22. *Mol. Cytogenet.* **2018**, *11*, 62.	NT = 11 mm High risk of Down syndrome
21.	22q11.21(18661724_21440514)x1	2.77 Mbp deletion	Male karyotype,the deletion covers the 22q11.2 deletion syndrome region (OMIM 188400) (ORPHA:567), including 52 protein-coding genes. Deletions of this region have been described in patients, among others, with heart defects, anatomical abnormalities of the palate, facial dysmorphic features, developmental delay, aplasia/hypoplasia of the thymus, and immunodeficiency.	NT = 1.2 mm HRHS,NB hypoplasia
22.	17p11.2(19143173_2021946	1.1 Mbp deletion	Female karyotype, Smith–Magenis syndrome (OMIM#182290) bez mutacji w RAI1. Vieira GH, Rodriguez JD, Carmona-Mora P, et al. Detection of classical 17p11.2 deletions, an atypical deletion and RAI1 alterations in patients with features suggestive of Smith–Magenis syndrome. *Eur. J. Hum. Genet.* **2012**, *20*, 148–154. doi:10.1038/ejhg.2011.167	NT = 1.3 mm Family history of mental retardation, Abnormal appearance of the eyelids after delivery
23.	16p11.2(29673954_30190568)x3	0.5 Mbp duplication	Female karyotype, chromosome 16p11.2 duplication syndrome (# 614671)	NT = 2.2 mm Intermediate risk of Down synfrome, parental request, FGR in third trimester+ hyperechogenic bowels
24.	1q43q44(242398564_249212668)x1	6.8 Mbp deletion	Male karyotype, chromosome 1q43-q44 deletion syndrome: mental retardation, autosomal dominant 22 (# 612337)	NT = 1.5 mm High risk of Down syndrome
25.	6q26q27(163373677_170921089)x1	7.56 Mbp deletion	Female karytype, 6q26 deletion syndrome	NT = 1.5 mm Amniocentesis due to Toxoplasma gondii infection
26.	22q11.21(18904835_21505400)x1	2.6 Mbp deletion	Male karyotype, the deletion covers the 22q11.2 deletion syndrome region (OMIM 188400) (ORPHA:567), including 52 protein-coding genes. Deletions of this region have been described in patients, among others, with heart defects, anatomical abnormalities of the palate, facial dysmorphic features, developmental delay, aplasia/hypoplasia of the thymus, and immunodeficiency.	NT = 1.6 mm IAA + VSD
27.	16p13.12p13.11(14762239_16194578)x1	1.4 Mbp deletion	Female karyotype, 16p13.11 microdeletion syndrome reported in UNIQUE database, https://www.rarechromo.org/media/information/Chromosome%2016/16p13.11%20microdeletions%20FTNW.pdf (accessed on 29 April 2024)	NT = 1.6 mm maternal request, AMA
28.	5p15.33p15.2(22149_10044258)x1	10 Mbp deletion	Female karyotype, CRI-DU-CHAT SYNDROME (# 123450)	NT = 1.7 mm DORV TOF type
29.	Xq28(149116213_155232907)x1	6.1 Mbp deletion	Male karyotype, Xq28 deletion syndrome in male (OMIM:# 300475)	NT = 1.7 mm RAA, thymus hypoplasia
30.	3q29(194969955_197317103)x1	2.35 Mbp deletion	Male karyotype, 3q29 DELETION SYNDROME (# 609425)	NT = 1.8 mm pulomoray valve dysplasia
31.	7p22.3p21.1(2634026_16758426)x3	14.1 Mbp duplcation	Male karyotype, 14.1 Mbp	NT = 4.7 mm Small kidneys, Oligohydramnion/amnioinfusion High risk of Down syndrome
32.	16p11.2(29673954_30190568)x1	0.5 Mbp deletion	Female karyotype, chromosome 16p11.2 deletion syndrome (# 611913)	NT = 4.8 mm carrier status in mother High risk of Down syndrome
33.	7q31.2q32.2(116255056_129694097)x3, 7q32.2q36.3(129853288_159085681)x1	13.4 Mbp duplication 29.2 Mbp deletion	Male karyotype; posssibly structural aberration in chromosome 7; duplication in UNIQUE database reported dup7q: https://www.rarechromo.org/media/information/Chromosome%20%207/7q%20Duplications%20FTNW.pdf (accessed on 29 April 2024) deletion: https://www.rarechromo.org/media/information/Chromosome%20%207/7q32q34%20deletions%20FTNW.pdf (accessed on 29 April 2024)	NT = 1.9 mm cleft palate, CHD, CCA
34.	18p11.32p11.21(148963_14081887)x1	13.9 Mbp deletion	Female karyotype, 18p deletions described at UniQue	NT = 1.9 mm holoprosencephaly
35.	1q21.1q21.2(145415190_147824207)x1	2.4 Mbp deletion	Female karyotype, chromosome 1q21.1 deletion syndrome (# 612474)	NT = 2 mm CHROMOSOME 1q21.1 DELETION SYNDROME in the mother and first son
36.	22q11.21(18894835_21505417)x1	2.6 Mbp deletion	Male karyotype, DiGeorge syndrome (OMIM#188400)	NT = 2.1 mm TOF + LSVC
37.	22q11.21q11.22(21808950_22905x1	1.1 Mbp deletion	Male karyotype, DiGeorge syndrome (OMIM#188400)	NT = 2.1 mm cleft lip and palate
38.	17p12p11.2(15801346_20464365)x1	4.66 Mbp deletion	Male karyotype, Smith–Magenis syndrome (OMIM#182290)	NT = 2.3 mm High risk od Down syndrome
39.	2p21(43391586_45394811)x1	2 Mbp deletion	Female karyotype, recurrent microdeletion 2q21.1: Report on a new patient with neurological disorders	NT = 2.3 mm semilobar holoprsonecephaly, cleft palate
40.	22q11.21q11.22(18894835_21505417)x1	2.6 Mbp deletion	Female karyotype, DiGeorge syndrome (OMIM#188400)	NT = 2.4 mm TOF + LSVC,thymus agenesis
41.	1q21.1q21.2(145415190_149275124)x3	3.86 Mbp duplication	Female, chromosome 1q21.1 duplication syndrome	NT = 2.4 mm High risk of Down syndrome, mental retardation in the mother
42.	2p16.3(51137071_51382872)x1	0.24 Mbp deletion	Male karyotype, chromosome 2p16.3 deletion syndrome (OMIM#614332)	NT = 2.4 mm DILV + PS
43.	22q11.1q11.21(17397498_20311763)x1	2.9 Mbp deletion	Male karyotype, DiGeorge syndrome (OMIM#188400) in corelation with translocation visible in GTG binding	NT = 2.5 mm Right isomerism
44.	5p15.33p15.1(22149_17479238)x1, 5p15.1p14.3(17745048_23253135)x4, 5p14.2p12(23503596_44506716)x3	17.46 Mbp deletion 5.51 Mbp duplication 21 Mbp duplication	Male karyotype, genome imbalance was found in the form of a deletion of the short arm of chromosome 5 in the 5p15.33p15.1 region with a size of ~17.46 Mbp, a triplication of the short arm of chromosome 5 in the 5p15.1p14.3 region with a size of ~5.51 Mbp and a duplication of the short arm of chromoIome 5 in the 5p14.2p12 region of ~21 Mbp. The 5p15.33p15.1 deletion covers the critical region of the 5p microdeletion syndrome (Cri Du Chat syndrome; OMIM: 123450).	NT = 2.7 mm AMA High risk of Down syndrome, AVSD
45.	1p36.33(779727_2063244)x1, 1p36.33p36.32(2162136_3444846)x3	1.28 Mbp deletion 1.28 Mbp duplication	Female karyotype, 1p36 microdeletion syndrome (OMIM# 607872; ORPHA:1606), 1p36 microduplication syndrome (ORPHA:96069)	NT = 4.7 mm early FGR High risk of Down syndrome
46.	6q25.2q27(153306440_170921089)x1, 20p13p12.3(60747_6317313)x3	17.6 Mbp deletion 6.26 Mpb duplication	Female karyotype, UNIQUE: 6q25.2q25.3 microdeletion syndrome (ORPHA:251056) and 6.26Mb duplication (UNIQUE: 20p duplication phenotype affected)	NT = 4.7 mm High risk of Down syndrome
47.	12p13.33p11.1(230421_34756209)x3	34.5 Mbp duplication	Female karyotype, Pallister–Killian syndrome (OMIM#601803)	NT = 2.8 mm CDH,flat profile, cleft palate, shortened bones, NB hypoplasia, FGR
48.	1q21.1q21.2(146155929_147824212)x1	1.67 Mbp deletion	Male karyotype, genome imbalance was found in the form of deletion of the long arm of chromosome 1 in the 1q21.1q21.2 region with a size of ~1.67 Mbp. The deletion covers the region of the known microdeletion syndrome 1q21.1 (OMIM: 612474)1 and belongs to pathogenic changes.	NT = 2.8 mm High risk of Down syndrome
49.	9p24.3p13.1(204090_38815471)x4	39 Mbp duplication	Male karyotype. Genomic imbalance was found in the form of tetrasomy of the short arm of chromosome 9 in the 9p24.3p13.1 region of ~39 Mbp. Chromosome analysis after cell culture (DM402/18) showed the presence of an additional isodicentric chromosome 9 in all 12 cells analyzed.	NT = 2.8 mm Age 36 years, hypoplastic nasal bone, posterior cranial fossa cyst, bilateral cleft lip, micrognathia, CHD.
50.	4p16.3p14(45889_38064734)x1	38 Mbp deletion	Female karyotype, genome imbalance was found in the form of deletion of the short arm of chromosome 4 in the 4p16.3p14 region with a size of ~38 Mbp.	NT = 4.8 mm Dysmorphic profile, CoA, club foot, abnormal first-trimester biochemistry High risk of Down syndrome
51.	11q24.1q25(123624201_134868407)x1	11.24 Mbp deletion	Female karyotype, Jacobsen syndrome (Deletion syndrome 11q, OMIM:# 147791	NT = 4.6 mm CHD High risk of Down syndrome
52.	22q11.22q11.23(22843461_25066484)x3dn	2.22 Mbp duplication	Male karyotype, genome imbalance was found in the form of a duplication of the long arm of chromosome 22 in the 22q11.22q11.23 region with a size of ~2.22 Mbp. The test result was confirmed by FISH (F2897). The duplIcation involves multiple genes, including the dose-sensitive SMARCB1 (OMIM: 6016071 and SNRPD3 (OMIM: 601062) genes.	NT = 3.1 mm history of fetal death, TR, VSD, NB hypoplasia
53.	7p14.3p14.1(32209242_41224388)x1	9 Mbp deletion	Male karyotype, genome imbalance was found in the form of a deletion of the short arm of chromosome 7 in the 7p14.3p14.1 region with a size of ~9 Mbp. The obtained test result confirms the 7p14 deletion found in the fetal karyotype test (study no. P 644/2017, Kraków)	NT = 3.2 mm High risk of Down syndrome
54.	9q21.13(77992580_78478835)x1	0.49 Mpb deletion	0,49Mb/possibly microdeletion 9q21.13 syndrome (ORPHA:531151)	NT = 3.3 mm High risk of Down syndrome
55.	22q11.23(23720181_25066484)x3,	1.35 Mbp duplication	Male karyotype, genome imbalance was found in the form of a duplication of the long arm of chromosome 22 in the 22q11.23 region with a size of ~1.35 Mbp. The dupIication involves multiple genes, including the dose-sensitive SMARCB1 (OMIM: 601607) and SNRPD3 (OMIM: 601062) genes. Duplications of this region may present with a wide range of clinical symptoms and may be inherited from parents who do not show clinical symptoms.	NT= 3.3 mm High risk of Down syndrome
56.	5p15.33p15.31(22149_7449397)x1, 5p15.31p12(7506131_44341490)x3	7.4 Mbp deletion duplication	Feamale karyotype, DELETION 5p15.3 is critical for Cri du Chat (OMIM#123450); DUPLICATION in UNIQUE reported trisomy 5p syndrome, phenotype depending on the size and location of the aberration https://www.crarechromo.Irg/media/information/Chromosome%20%205/Trisomy%205p%20Duplications%20of%205p15%20FTNW.pdf (accessed on 29 April 2024)	NT = 3.4 mm PA + VSD, FGR, NB hypoplasia
57.	2q13(110862477_111368891)x1, 12p13.33p13.32(23.421_3358023)x1	0.5 Mb deletion 3.1 Mbp deletion	0.5Mb (2q13 benign variant), 3.1Mb (12p deletion pathogenic-12p13.33 deletion syndrome (ORPHA:280325)	NT = 3.4 mm hemivertebra, cerebellum hypoplasia, kidney dysplasia, club foot
58.	20p12.3(7246510_8558204)x1	1.31 Mbp deletion	Female karyotype, genome imbalance was found in the form of a deletion of the short arm of chromosome 20 in the 20p12.3 region with a size of ~1.31 Mbp. Examination of the parents using aCGH method showed that the deletion detected in the fetus was of paternal origin. The deletion includes the following genes: HAO1 (OMIM 605023)1, TMX4 (OMIM 616766)1, and exons 1–3 of the dose-sensitive gene PLCB1 (OMIM 607120)1.	NT = 3.5 mm High risk of Down syndrome
59.	22q11.21(18894820_21457610)x1	2.56 Mbp deletion	Female karyotype, genome imbalance was found in the form of an interstitial deletion of the long arm of chromosome 22 in the 22q11.21 region with a size of ~2.56 Mbp. The deletion covers the 22q11.2 deletion syndrome region (OMIM 188400)1 (ORPHA:567)2, including 52 protein-coding genes. Deletions of this region have been described in patients, among others, with heart defects, anatomical abnormalities of the palate, facial dysmorphic features, developmental delay, thymic aplasia/hypoplasia, and immunodeficiency. Aberration is a pathogenic change.	NT = 3.5 mm High risk of Down syndrome
60.	16p13.11(15048732_16194575)x1,(21)x3	1.15 Mbp deletion	Male karyotype, additionally, an interstitial deletion of the short arm of chromosome 16 was found in the 16p13.11 region with a size of ~1.15 Mbp. The aberration involves the 16p13.11 microdeletion syndrome region (ORPHA:261236)1, including 11 protein-coding genes. 16p13.11 deletions have been described in patients mainly with neurodevelopmental disorders (psychomotor development delay, epilepsy, behavioral disorders), microcephaly, short stature, and less frequently with dysmorphic features and congenital defects. The aberration is a potentially pathogenic lesion, characterized by variable expression and incomplete penetrance (13.1%)3 and may be inherited from parents who do not show clinical symptoms. Despite the low penetrance, the aberration was included in the test result due to the presence of trisomy of chromosome 21 of the pair.	NT = 3.6 mm AMA High risk of Down syndrome
61.	17p12(14111772_15442066)x1	1.33 Mbp deletion	Female karyotype, neuropathy, hereditary, with liability to pressure palsies; HNPP(OMIM#162500)	NT = 3.7 mm High risk of Down syndrome
62.	22q11.21(18894820_21457610)x3	2.56 Mbp duplication	Female karyotype, genome imbalance was found in the form of a duplication of the long arm of chromosome 22 in the 22q11.21 region with a size of ~2.56 Mbp. The duplication covers the region of the known microduplication complex 22q11.2 (OMIM: 608363)1.	NT = 3.9 mm AMA, High risk of Down syndrome,
63.	5p15.33p14.1(22149_27788732)x1, 13q31.2q34(89267673_115093115)x3	28 Mbp deletion 26 Mbp duplication	Male karyotype, genome imbalance was found in the form of a deletion of the short arm of chromosome 5 in the 5p15.33p14.1 region of ~28 Mbp and a duplication of the long arm of chromosome 13 in the 13q31.2q34 region of ~26 Mbp. The detected aberrations belong to pathogenic changes. The 5p15.33p14.1 deletion affects 67 protein-coding genes and the 5p deletion syndrome region (OMIM 123450). Patients with this syndrome have been described, among others, with IUGR, characteristic crying, hypotonia, microcephaly, dysmorphic facial features, delay in psychomotor development and intellectual disability. The 13q31.2q34 duplication covers 87 protein-coding genes. Clinical features found in people with distal duplication of chromosome 13q (ORPHA 96105) include: developmental delay, intellectual disability, behavioral disorders, facial dysmorphia, and congenital defects of the nervous, cardiovascular and urogenital systems.	NT = 3.9 mm High risk of Down syndrome
64.	22q11.21(18765102_20311733)x3,mat	1.55 Mbp duplication	Female karyotype, genome imbalance was found in the form of a duplication of the long arm of chromosome 22 in the 22q11.21 region with a size of ~1.55 Mbp. The aberration partially involves the region of the known microduplication complex 22q11.2 (OMIM: 608363).	NT = 4.1 mm High risk of Down syndrome
65.	20p11.23p11.21(19852522_22840889)x1	2.99 Mbp deletion	Male karyotype, proximal deletion 20p	NT = 5.2 mm left isomerism,bradycardia
66.	5p15.33p13.1(22149_40841558)x3, 9p24.3p24.2(204090_2267859)x1	41 Mbp duplication 2 Mbp deletion	Female karyotype, genome imbalance was found in the form of a duplication of the short arm of chromosome 5 in the 5p15.33p13.1 region of ~41 Mbp and a deletion of the short arm of the chromosome 9 in the 9p24.3p24.2 region of ~2 Mb.	NT = 4.2 mm High risk of Down syndrome
67.	3p26.3p26.1(127472_5179975)x3, 6p25.3p25.1(164360_5733668)x1	5.05 Mbp duplication 5.57 Mbp deletion	Female karyotype, genome imbalance was found in the form of a duplication of the short arm of chromosome 3 in the 3p26.3p26.1 region of ~5.05 Mbp and a deletion of the short arm of chromosome 6 in the 6p25.3p25.1 region of ~5.57 Mbp. The test result was confirmed by FISH (study no. F2937).	NT = 4.2 mm AMA, HLHS High risk of Down syndrome
68.	20q13.33(62103563_62908679)x1~2	0.805 Mbp deletion	Male karyotype, genome imbalance was found in the form of a mosaic deletion of the long arm of chromosome 20 in the 20q13.33 region with a size of ~805 kb. The deletion covers 33 protein-coding genes, including exon 1 of the dose-sensitive KCNQ2 gene (OMIM 602235)1 and the following genes: EEF1A2 (OMIM 602959)1, RTEL1 (OMIM 608833)1, DNAJC5 (OMIM 611203)1, PRPF6 (OMIM 613979) 1, and SOX18 (OMIM 601618)1. Aberration is a potentially pathogenic change.	NT = 4.3 mm AMA High risk of Down syndrome
69.	8q11.1q11.21(47456485_51757184)x3	4.3 Mbp duplication	Female karyotype, in UNIQUE database reported 8q duplication syndrome: https://www.rarechromo.org/media/information/Chromosome%20%208/8q%20duplications%20FTNW.pdf (accessed on 29 April 2024)	NT = 4.4 mm High risk of Down syndrome
70.	Xp21.1(32006239_32383121)x0	377 kb deletion	Male karyotype, genome imbalance was found in the form of a deletion of the long arm of the X chromosome in the Xp21.1 region of ~377 kb. The deletion covers exons 36–44 of the DMD gene (OMIM 300377)1. Mutations and deletions of this gene have been described in patients with Duchenne Muscular Dystrophy (OMIM: 310200)1 and Becker Muscular Dystrophy (OMIM: 300376)1 and may be inherited from mothers, carriers who do not show clinical symptoms	NT = 4.4 mm High risk of Down syndrome
71.	22q11.21(18894820_21457610)x3	2.56 Mbp duplication	Male karyotype, genome imbalance was found in the form of a duplication of the long arm of chromosome 22 in the 22q11.21 region with a size of ~2.56 Mbp. The duplication covers the region of the known microduplication complex 22q11.2 (OMIM: 608363)1. Duplications of this region may present with a wide range of clinical features and may be inherited from asymptomatic parents.	NT = 4.4 mm High risk of Down syndrome
72.	Xp22.31(7145359_7159518)x0	0.01 Mb deletion	Male karyotype, Ichthyosis, X-linked (OMIM#308100)	NT = 4.5 mm High risk of Down syndrome
73.	1p33p32.3(48016425_52280457)x1	4.26 Mb deletion	Female karyotype, genome imbalance was found in the form of deletion of the short arm of chromosome 1 in the 1p33p32.3 region with a size of ~4.26 Mbp The deletion covers 15 protein-coding genes, including dose-sensitive genes: AGBL4 (OMIM 616476)1, ELAVL4 (OMIM 168360)1, FAF1 (OMIM 604460)1, CDKN2C (OMIM 603369)1, RNF11 (OMIM 612598)1, EPS15 (OMIM 600051).	NT = 4.5 mm High risk of Down syndrome
74.	5p15.33p15.32(22149_4768822)x1, 5p15.2(10212960_12513658)x1, 11p15.5p14.1(113082_27880946)x3	4.75 Mbp deletion 2.3 Mbp deletion 27.77 Mbp duplication	Male karyotype, genome imbalance was found in the form of a terminal deletion of the short arm of chromosome 5 in the region 5p15.33p15.32 with a size of ~4.75 Mbp and an interstitial deletion of the short arm of chromosome 5 in the region 5p15.2 with a size of ~2.3 Mbp and terminal duplication of the short arm of chromosome 11 in the 11p15.5p14.1 region with a size of ~27.77 Mbp. The 5p15.33p15.32 deletion covers 29 protein-coding genes, including the following genes: SDHA (OMIM 600857)1, SLC9A3 (OMIM 182307)1, TRIP13 (OMIM 604507)1, SLC6A19 (OMIM 608893)1, TERT (OMIM 187270)1, SLC6A3 (OMIM 126455)1, and NDUFS6 (OMIM 603848)1. The 5p15.2 deletion covers 8 protein-coding genes, including CCT5 (OMIM 610150)1 and MARCH6 (OMIM 613297)1. The detected deletions are located in the region of Cri du Chat syndrome (OMIM 123450)1, described in patients including those with microcephaly, growth retardation, characteristic crying in the neonatal/infantile period, dysmorphic facial features, and significant delay in psychomotor development. The duplication in the 11p15.5p14.1 region covers 314 protein-coding genes, as well as the region of Beckwith–Wiedemann syndrome (OMIM 130650)1, described in patients including those with excessive growth, predisposition to cancer and congenital defects, and the region of Silver–Russell syndrome (OMIM 180860)1 described in patients, among others, with short stature, limb asymmetry, and facial dysmorphic features. The detected aberrations belong to pathogenic changes.	NT = 4.65 mm, Hig risk of Down syndrome
75.	14q32.12q32.33(93833237_107287708)x1	13.45 Mbp deletion	Female karyotype, genome imbalance was found in the form of a terminal deletion of the long arm of chromosome 14 in the 14q32.12q32.33 region with a size of ~13.45 Mbp. The aberration affects 201 protein-coding genes, including dose-sensitive genes: DICER1 (OMIM 606241)1, PAPOLA (OMIM 605553)1, YY1 (OMIM 600013)1, HSP90AA1 (OMIM 140571)1, WDR20 (OMIM 617741)1, EIF5 (OMIM 601710)1, and AKT1 (OMIM 164730)1. The deletion covers the region of 14q32 deletion syndrome (IDDHDF, OMIM 618147)1, described in patients including those with developmental delay, hypertelorism, and facial dysmorphic features. Terminal deletions in the 14q32 region have been described in patients with, among others, growth retardation, hypotonia, dysmorphic features, intellectual disability, developmental delay, and heart defects. Aberration is a pathogenic change.	NT = 4.7 mm CHD(VSD), DV PI = 1.4, High risk of Down syndrome
76.	22q11.21(18628147_21661435)x1	3.03 Mbp deletion	Female karyotype, genome imbalance was found in the form of an interstitial deletion of the long arm of chromosome 22 in the 22q11.21 region with a size of ~3.03 Mbp. The deletion covers the 22q11.2 deletion syndrome region (OMIM 188400)1 (ORPHA:567)2, including 52 protein-coding genes. Deletions of this region have been described in patients, among others, with heart defects, anatomical abnormalities of the palate, facial dysmorphic features, developmental delay, thymic aplasia/hypoplasia, and immunodeficiency1,2.Aberration is a pathogenic change.	NT = 4.7 mm High risk of Down syndrome
77.	22q11.21(18894820_21457610)x1	2.56 Mbp deletion	Female karyotype, genome imbalance was found in the form of an interstitial deletion of the long arm of chromosome 22 in the 22q11.21 region with a size of ~2.56 Mbp. The deletion covers the 22q11.2 deletion syndrome region (OMIM 188400)1 (ORPHA:567)2, including 52 protein-coding genes. Deletions of this region have been described in patients, among others, with heart defects, anatomical abnormalities of the palate, facial dysmorphic features, developmental delay, thymic aplasia/hypoplasia, and immunodeficiency.Aberration is a pathogenic change.	NT = 4.7 mm TOF High risk of Down syndrome
78.	22q11.21(18628147_21759580)x1dn	3.13 Mbp deletion	Female karyotype, genome imbalance was found in the form of a deletion of the long arm of chromosome 22 in the 22q11.21 region with a size of ~ 3.13 Mbp. The deletion covers the region of the known microdeletion syndrome 22q11.2 (OMIM: 188400)1 and is responsible for for abnormalities detected in the fetus during ultrasound examination.	NT = 4.8 mm TOF High risk of Down syndrome
79.	2p21(44507916_44618832)x1, 12p13.33p13.1(244335_13244974)x3	110 kb deletion 13 Mbp duplication	Female karyotype, genome imbalance was found in the form of a deletion of the short arm of chromosome 2 in the 2p21 region of ~110 kb and a duplication of the short arm of chromosome 12 in the 12p13.33p13.1 region of ~13 Mb. The obtained test result indicates that the additional material on chromosome 12 found in the karyotype study comes from the short arm of chromosome 12 (region 12p13.33p13.1). Since karyotypes of the parents were previously performed (outside IMiD), a familial origin of the 12p13.33p13.1 duplication can be ruled out with high probability. However, the 2p21 deletion covers the dose-sensitive PREPL gene (OMIM: 609557)1, exons 2–10 of the SLC3A1 gene (OMIM: 104614)1 and exons 1–3 of the CAMKMT gene (OMIM: 609559)1 and is located in the region of the known 2p21 microdeletion syndrome (OMIM: 606407).	NT = 4.8 mm High risk of Down syndrome
80.	Xp22.31(7226972_7679167)x0	0.45 Mbp deletion	Male karyotype, Ichthyosis, X-linked (OMIM#308100)	NT = 5.5 mm megacystis, NB hypoplasia High risk of Down syndrome
81.	2q13(111646676_113065741)x1mat	1.42 Mbp deletion	Female karyotype, genome imbalance was found in the form of deletion of the long arm of chromosome 2 in the 2q13 region with a size of ~1.42 Mbp The 2q13 deletion includes the following genes: BCL2L11 (OMIM 603827)1, ANAPC1 (OMIM 608473)1, MERTK (OMIM 604705)1, FBLN7 (OMIM 611551)1, and exons 1–3 of the dose-sensitive ZC3H62. Deletions of this region have been described in patients with heart defects, dysmorphic features, abnormal head circumference, hypotonia, and in some cases also with intellectual disability and autism spectrum disorders. These deletions may be inherited from parents who do not show clinical symptoms. Deletion is a potentially pathogenic change.	NT = 5.7 mm Small head, TR, VSD, abnormal profile High risk of Down syndrome
82.	3q23q29(141143997_197837049)x3	56.7 Mbp duplication	Male karyotype	NT = 5.9 mm DORV High risk of Down syndrome
83.	10q26.13q26.3(123902691_135404550)x1, 11q23.3q25(120610827_134868420)x3, Xp22.12(19363761_20185740)x2	11.5 Mbp deletion 14.26 Mbp duplication 822 kp duplication	Male karyotype, genome imbalance in the form of deletion of the long arm of chromosome 10 in the 10q26.13q26.3 region of ~11.5 Mbp, and duplication of the long arm of the chromosome 11 in the 11q23.3q25 region with a size of ~14.26 Mbp. Additionally, the study found a duplication of the short arm of the X chromosome in the Xp22.12 region With a size of ~822 kb. This duplication includes the following genes: PDHA1 (OMIM: 300502)1, MAP3K16 (OMIM: 300820)1, SH3KBP1 (OMIM: 300374)1, MAP7D22, and exons 17–22 of the dose-sensitive RPS6KA3 gene (OMIM: 300075)1. Duplications in the RPS6KA3 gene have been described in patients with Coffin–Lowry syndrome (OMIM: 303600)1.	NT = 6.6 mm cystic hygroma, abnormal profile, AMA High risk of Down syndrome
84.	1p36.32p35.3(2558854_29403494)x3	26.8 Mbp duplication	Chromosome 1p36.33 duplication syndrome, ATAD3 gene cluster (OMIM#618815)	NT = 7.5 mm hygroma colli
85.	2p25.3p24.3(21191_13382780)x3, 4p16.3p15.31(45889_20673992)x1	13.36 Mbp duplication 20.63 Mbp deletion	Male karyotype, genome imbalance was found in the form of a duplication of the short arm of chromosome 2 in the 2p25.3p24.3 region of ~13.36 Mbp and a deletion of the short arm of chromosome 4 in the 4p16.3p15.31 region of ~20.63 Mbp.	NT = 7.7 mm High risk of Down syndrome
86.	Xq25q28(126504430_152689544)x3, Xq28(152788476_155246643)x1	26.19 Mbp duplication 2.46 Mbp deletion	Female karyotype, genome imbalance was found in the form of a duplication of the long arm of the X chromosome in the Xq25q28 region with a size of ~26.19 Mbp and a deletion of the long arm of the X chromosome in the Xq28 region with a size of ~2.46 Mbp.	NT = 8.4 mm Hygroma colli High risk of Down syndrome
87.	1q43q44(238724105_249203359)x3 dn, 18q22.3q23(70759329_78005754)x1 dn	10.48 Mbp duplication 7.25 Mbp deletion	Female karyotype, genome imbalance was found in the form of a duplication of the long arm of chromosome 1 in the 1q43q44 region of ~10.48 Mbp and a deletion of the long arm of chromosome 18 in the 18q22.3q23 region of ~7.25 Mbp.	NT = 8.8 mm High risk of Down syndrome

aCGH, array comparative genomic hybridization; AMA, advanced maternal age; ASD, atrial septal defect; AVSD, atrioventricular septal defect; CCA, corpus callosum agenesis; CDH, congenital diaphragmatic hernia; CHD, congenital heart defect; CNV, copy number variant; CoA, coarctation of the aorta; DILV, double inlet ventricle; DORV; double outlet right ventricle; DV, ductus venosus; FGR, fetal growth restriction; HLHS, hypoplastic left heart syndrome; HRHS, hypoplastic right heart syndrome; IAA, interrupted aortic arch; LSVC, left superior vena cava; NB, nasal bone; NT, nuchal translucency; PA, pulmonary atresia; PI, pulsatility index; PS, pulmonary stenosis; RAA, right aortic arch; TOF, tetralogy of Fallot; TR, tricuspid regurgitation; VSD, ventricular septum defect.

**Table 3:** Frequency of chromosomal aberrations in classical karyotype and CNVs detected by aCGH depending on the nuchal translucency (NT) value.

	NT < p95 n = 754	NT p95-2.9 mm n = 142	NT 3.0–3.4 mm n = 229	NT 3.5–3.9 mm n = 139	NT 4.0–4.5 mm n = 115	NT > 4.5 mm n = 367
**Numerical chromosomal aberrations, n (%)**	**45 (5.9)**	**24 (16.9)**	**43 (18.7)**	**32 (23.0)**	**34 (29.5)**	**159 (43.3)**
**CNVs, n (%)**	**22 (3.0)**	**4 (2.8)**	**8 (3.5)**	**6 (4.3)**	**14 (12.2)**	**33 (9.0)**
**CNVs in fetuses with normal karyotype, (%)**	**3.1%**	**3.3%**	**4.3%**	**5.6%**	**17.2%**	**15.8%**

aCGH, array comparative genomic hybridization; CNVs, copy number variants; NT, nuchal translucency.

**Table 4:** Detected chromosomal aberrations depending on the nuchal translucency (NT) value and associated anomalies.

With Associated Anomalies	NT < p95 N = 300	NT p95-2.9 mm N = 68	NT 3.0–3.4 mm N = 42	NT 3.5–3.9 mm N = 22	NT 4.0–4.5 mm N = 27	NT > 4.5 mm N = 175
**All pathogenic chromosomal aberrations, n (%)**	**43 (14.3)**	**16 (23.5)**	**14 (33.3)**	**8 (36.3)**	**11 (40.7)**	**103 (58.8)**
**Numerical chromosomal aberrations, n (%)**	**30 (10.0)**	**13 (19.1)**	**8 (19.0)**	**7 (31.8)**	**9 (33.3)**	**88 (50.2)**
T21, n (%)	8 (2.6)	9 (13.2)	0 (0.0)	2 (9.0)	8 (29.6)	27 (15.4)
T18, n (%)	7 (2.3)	1 (1.4)	4 (9.5)	3 (13.6)	1 (3.7)	23 (13.1)
T13, n (%)	5 (1.6)	0 (0.0)	2 (4.7)	1 (4.5)	0 (0.0)	11 (6.3)
45XO, n (%)	1 (0.3)	1 (1.4)	1 (2.4)	0 (0.0)	0 (0.0)	27 (15.4)
47,XXX; 47,XXY, n (%)	1 (0.3)	1 (1.4)	0 (0.0)	0 (0.0)	0 (0.0)	0 (0.0)
**Triploidy, n (%)**	**8 (2.6)**	**1 (1.4)**	**1 (1.4)**	**1 (1.4)**	**0 (0.0)**	**0 (0.0)**
**CNVs, n (%)**	**13 (4.3)**	**3 (4.4)**	**6 (14.3)**	**1 (4.5)**	**2 (7.4)**	**15 (8.6)**
**CNVs in fetuses with normal karyotype, (%)**	**4.8**	**5.4**	**17.6**	**6.6**	**11.1**	**17.2**
**Without associated anomalies, n**	**NT < p95** **N = 454**	**NT p95-2.9 mm** **N = 74**	**NT 3.0–3.4 mm** **N = 187**	**NT 3.5–3.9 mm** **N = 117**	**NT 4.0–4.5 mm** **N = 88**	**NT > 4.5 mm** **N = 192**
**All pathogenic chromosomal aberrations, n (%)**	**24 (5.3)**	**12 (16.2)**	**37 (19.8)**	**30 (25.6)**	**37 (42.0)**	**89 (46.3)**
**Numerical chromosomal aberrations, n (%)**	**15 (3.3)**	**11 (14.8)**	**35 (18.7)**	**25 (21.3)**	**25 (28.4)**	**71 (36.9)**
T21, n (%)	9 (2.0)	7 (9.4)	31 (16.6)	19 (16.2)	20 (22.7)	35 (18.2)
T18, n (%)	0 (0.0)	0 (0.0)	1 (0.5)	3 (2.5)	1 (1.1)	13 (6.7)
T13, n (%)	0 (0.0)	0 (0.0)	0 (0.0)	0 (0.0)	2 (2.2)	3 (1.5)
45X0, n (%)	2 (0.4)	0 (0.0)	3 (1.5)	3 (2.5)	2 (2.2)	19 (9.9)
47,XXX; 47,XXY, n (%)	4 (0.8)	3 (4.0)	0 (0.0)	0 (0.0)	0 (0.0)	1 (0.5)
**Triploidy, n (%)**	**0 (0.0)**	**0**	**0**	**0**	**0**	**0**
**CNVs, n (%)**	**9 (2.0)**	**1 (1.3)**	**2 (1.0)**	**5 (4.3)**	**12 (13.6)**	**18 (9.3)**
**CNVs in fetuses with normal karyotype, (%)**	**2.0**	**1.5**	**1.3**	**5.4**	**19.0**	**14.8**

aCGH, array comparative genomic hybridization; NT, nuchal translucency; CNVs, copy number variants; T13, trisomy 13; T18, trisomy 18; T21, trisomy 21.

## Data Availability

The raw data supporting the conclusions of this article will be made available by the authors upon request.

## Abbreviations

aCGH: array comparative genomic hybridization; cfDNA, cell-free DNA; CNV, copy number variant; CRL, crown–rump length; NT, nuchal translucency.
